# Bimodal Incoherent Digital Holography for Both Three-Dimensional Imaging and Quasi-Infinite–Depth-of-Field Imaging

**DOI:** 10.1038/s41598-019-39728-8

**Published:** 2019-03-04

**Authors:** Teruyoshi Nobukawa, Yutaro Katano, Tetsuhiko Muroi, Nobuhiro Kinoshita, Norihiko Ishii

**Affiliations:** Science & Technology Research Laboratories, Japan Broadcasting Corporation (NHK), Kinuta 1-10-11, Setagaya, Tokyo 157-8510 Japan

## Abstract

Although three-dimensional (3D) imaging and extended depth-of-field (DOF) imaging are completely opposite techniques, both provide much more information about 3D scenes and objects than does traditional two-dimensional imaging. Therefore, these imaging techniques strongly influence a wide variety of applications, such as broadcasting, entertainment, metrology, security and biology. In the present work, we derive a generalised theory involving incoherent digital holography to describe both 3D imaging and quasi-infinite–DOF (QIDOF) imaging, which allows us to comprehensively discuss the functions of each imaging technique. On the basis of this theory, we propose and develop a bimodal incoherent digital holography system that allows both 3D imaging and QIDOF imaging. The proposed system allows imaging objects using spatially incoherent light and reconstructing 3D images or QIDOF images solely by changing the phase pattern of a spatial light modulator and without requiring mechanical adjustments or any other modifications to the setup. As a proof-of-principle experiment, we evaluate the DOF and record holograms of a reflective object with the proposed system. The experimental results show that the generalised theory is effective; our demonstration platform provides the function of 3D and QIDOF imaging.

## Introduction

Three-dimensional (3D) imaging can provide significantly more information about real 3D scenes and objects than can traditional two-dimensional (2D) imaging with a camera. Although it is yet relatively unknown, 3D imaging is gradually becoming ubiquitous in a wide variety of applications such as broadcasting, entertainment, metrology, security and biology and has been studied extensively in both industry and academia. In addition to 3D imaging, extended depth-of-field (DOF) imaging can also provide significant information about 3D scenes and objects. Although depth information on 3D objects is lost, extended-DOF imaging provides focused 2D images of 3D objects on different axial planes. Extended-DOF imaging offers a quick and precise overview of the 2D shape projected by a 3D object. Therefore, 3D imaging and extended-DOF imaging exploit the DOF in completely opposite ways, yet both are useful in observing real 3D scenes and objects. In what follows, we develop a system that allows both 3D imaging and extended-DOF imaging.

Numerous approaches have been proposed for 3D imaging, such as stereo vision, depth from defocus, time of flight and integral imaging. Among these techniques, digital holography is one of the very attractive techniques for 3D imaging because it captures and views a 3D volume by detecting the complex-amplitude distribution^1^. Digital holography generally requires a coherent light source, such as a laser, to form holograms, which leads to technical difficulties in practical use and thereby restricts its applications. However, incoherent digital holography^2,3^ eliminates the coherence requirement; instead, it is based on a passive recording process and can form holograms using a spatially incoherent light source, such as sunlight, fluorescence or light-emitting diodes (LEDs). Moreover, because it violates the Lagrange invariant law, which describes the relationship between the numerical apertures of an object and an image, incoherent digital holography produces images with higher spatial resolution than what is possible with traditional 2D imaging^4,5^.

Although reducing the numerical aperture of an imaging lens is a straightforward method to implement extended-DOF imaging, it sacrifices light throughput and spatial resolution. To overcome this problem, focal stack and wave-front coding^6–8^ have been proposed. Interestingly, extended-DOF imaging can also be implemented using incoherent digital holography^9^. From a theoretical viewpoint, the DOF range of incoherent digital holography is quasi-infinite provided that a detectable hologram forms on the detector^10,11^. We refer to extended-DOF imaging of incoherent digital holography as quasi-infinite–DOF (QIDOF). QIDOF imaging also offers higher spatial resolution than traditional 2D imaging does^12^.

As mentioned above, incoherent digital holography is applicable to both 3D and QIDOF imaging and offers high spatial resolution for both imaging techniques. Given a platform that enables 3D and QIDOF imaging with incoherent digital holography, 3D scenes or objects could be observed from a different point of view, which would allow moving 3D objects to be tracked. Moreover, both 3D and QIDOF imaging offer a new means of expression in photography and videography by providing images that are much more informative than those obtained with a traditional 2D camera. However, to date, these two imaging techniques for incoherent digital holography have been developed individually, so no fundamental theory exists for simultaneously implementing both of them with incoherent digital holography.

Thus, here, we developed a general analytical theory that describes 3D and QIDOF imaging with incoherent digital holography. This theory elucidates the imaging properties of incoherent digital holography such as resolution, field of view, DOF and telecentricity conditions. Moreover, on the basis of this theory, we proposed and developed a bimodal incoherent digital holography system that allows both 3D and QIDOF imaging.

This article is organised as follows. First, based on wave propagation, we theoretically describe the generalised principle of bimodal incoherent holography. To verify the pertinence of the derived equation, we compare it with previously reported equations. Next, we theoretically describe the telecentricity conditions for 3D and QIDOF imaging and show that the telecentricity conditions differ for each imaging technique. After that, we experimentally evaluate the DOF for each imaging technique and compare it with the DOF of a traditional 2D imaging system to verify the capability of the proposed method. Finally, we experimentally demonstrate the proposed bimodal incoherent digital holography system by using it to image a reflective object.

## Principle of Bimodal Incoherent Digital Holography

**Figure 1:**
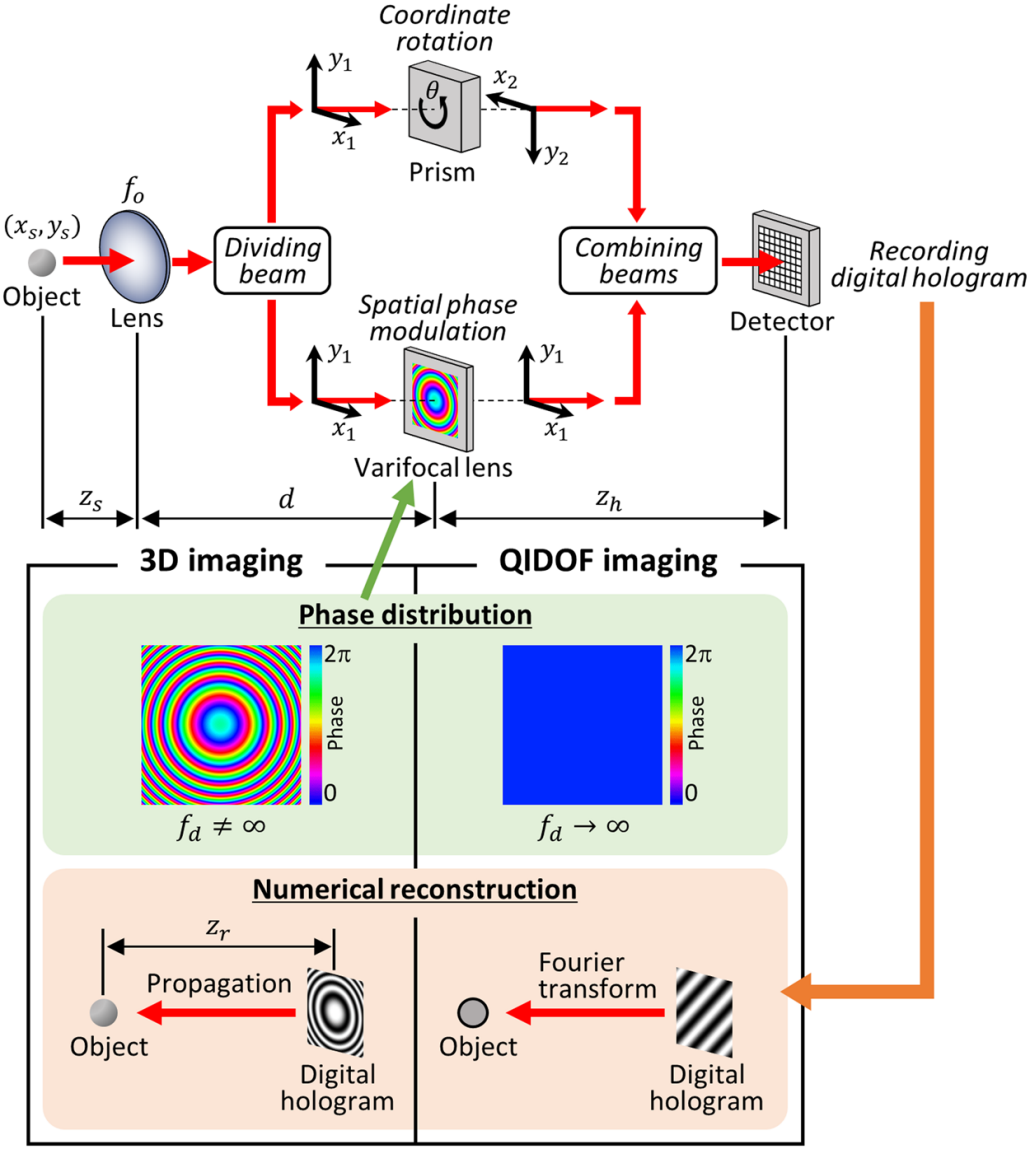
A schematic of bimodal incoherent digital holography for both of 3D and QIDOF imaging.

Figure 1 shows a schematic of a bimodal incoherent holography system that allows both 3D and QIDOF imaging. Unlike traditional holography with a coherent light source^1^, the proposed system does not discriminate between the object and the reference beams. The recording process is entirely based on the self-interference of a spatially incoherent light source. A self-emitting 3D object or one illuminated by a spatially incoherent light source can be regarded as a collection of many point sources. For the sake of simplicity, we describe the recording and reconstruction processes for a single point source located at ${\overrightarrow{r}}_{s}=({x}_{s},\,{y}_{s})$. As shown in Fig. 1, a beam of wavelength *λ* from the single point source propagates a distance *z*_*s*_ and is incident on a lens of focal length *f*_*o*_. Although this lens is optional, it is introduced to allow for adjusting the image magnification and field of view and to collect the light energy. Based on Fresnel diffraction, the complex amplitude *u*(*x*_1_, *y*_1_) in a plane immediately behind the lens is

1 $$u({x}_{1},{y}_{1})={Q}_{1}[\frac{{f}_{o}-{z}_{s}}{{z}_{s}\,{f}_{o}}]{L}_{1}[\frac{-1}{{z}_{s}}{\overrightarrow{r}}_{s}],$$

where *Q*_1_ and *L*_1_ are the quadratic and linear phase distributions, respectively, and are defined as $${Q}_{1}[A]=\exp $$$$[i\pi A{\lambda }^{-1}({x}_{1}^{2}+{y}_{1}^{2})]$$ and *L*_1_[$$\overrightarrow{A}$$] = exp[*i*2*πλ*^−1^(*A*_*x*_*x*_1_ + *A*_*y*_*y*_1_)]. The spatial coordinates are (*x*_1_, *y*_1_). Note that the constant term is omitted for brevity. This beam is divided into two copies, one of which propagates over a distance *d*. The spatial phase distribution of the propagated beam is modulated using a varifocal lens. The modulated beam propagates to a detector positioned at a distance *z*_*h*_ from the varifocal lens. By convoluting the Fresnel diffraction, the complex amplitude *u*_1_(*x*_1_, *y*_1_) on the detector may be represented as

2 $${u}_{1}({x}_{1},{y}_{1})={Q}_{1}[\frac{{f}_{d}-{z}_{d}}{{z}_{d}\,{f}_{d}+{z}_{h}\,{f}_{d}-{z}_{d}{z}_{h}}]{L}_{1}[\frac{-{f}_{o}{f}_{d}}{({f}_{o}-{z}_{s})({z}_{d}\,{f}_{d}+{z}_{h}\,{f}_{d}-{z}_{d}{z}_{h})}{\overrightarrow{r}}_{s}],$$

where *f*_*d*_ is the focal length of the varifocal lens that determines the function of 3D and QIDOF imaging and

3 $${z}_{d}=\frac{{z}_{s}\,{f}_{o}+d{f}_{o}-d{z}_{s}}{{f}_{o}-{z}_{s}}.$$

In contrast, the other beam copy propagates a distance *d* + *z*_*h*_ with no spatial phase modulation. On the way to the detector, the spatial coordinates of this beam are rotated by an angle *θ*. The rotated coordinates (*x*_2_, *y*_2_) are given by

4 $$(\begin{array}{c}{x}_{2}\\ {y}_{2}\end{array})=(\begin{array}{cc}\cos \,\theta  & -\sin \,\theta \\ \sin \,\theta  & \cos \,\theta \end{array})(\begin{array}{c}{x}_{1}\\ {y}_{1}\end{array})={\boldsymbol{R}}(\theta )(\begin{array}{c}{x}_{1}\\ {y}_{1}\end{array}),$$

where ***R***(*θ*) is the rotation matrix. Given the coordinates (*x*_2_, *y*_2_) and the Fresnel diffraction, the complex amplitude *u*_2_(*x*_2_, *y*_2_) of the rotated beam on the detector is

5 $${u}_{2}({x}_{2},{y}_{2})={Q}_{2}[\frac{1}{{z}_{d}+{z}_{h}}]{L}_{2}[\frac{-{f}_{o}}{({f}_{o}-{z}_{s})({z}_{d}+{z}_{h})}{\overrightarrow{r}}_{s}],$$

where ${Q}_{2}[A]=\exp [i\pi A{\lambda }^{-1}({x}_{2}^{2}+{y}_{2}^{2})]$ and *L*_2_[$\overrightarrow{A}$] = exp[*i*2*πλ*^−1^(*A*_*x*_*x*_2_ + *A*_*y*_*y*_2_)]. The two beams given by Eqs (2) and (5) interfere with each other on the detector, resulting in the interference pattern

6 $$I={|{u}_{1}|}^{2}+{|{u}_{2}|}^{2}+{u}_{1}{u}_{2}^{\ast }+{u}_{1}^{\ast }{u}_{2}.$$

The detector captures the interference pattern as a digital hologram. This digital hologram consists of a bias |*u*_1_|^2^ + |*u*_2_|^2^ and a twin image ${u}_{1}^{\ast }{u}_{2}$, in addition to the desired component ${u}_{1}{u}_{2}^{\ast }$. The presence of the bias and the twin image terms generally degrades the reconstructed images. To remove these undesirable components, researchers have used the phase-shifting method^2,3,13–17^, the off-axis technique^18–21^ or compressive sensing^22^. Once the undesirable components are removed, the desired complex amplitude of the digital hologram may be extracted as follows:

7 $$\begin{array}{ccc}{u}_{1}{u}_{2}^{\ast } & = & {Q}_{1}[\frac{{f}_{d}-{z}_{d}}{{z}_{d}\,{f}_{d}+{z}_{h}\,{f}_{d}-{z}_{d}{z}_{h}}]{L}_{1}[\frac{-{f}_{o}{f}_{d}}{({f}_{o}-{z}_{s})({z}_{d}\,{f}_{d}+{z}_{h}\,{f}_{d}-{z}_{d}{z}_{h})}{\overrightarrow{r}}_{s}]{Q}_{2}[\frac{-1}{{z}_{d}+{z}_{h}}]{L}_{2}[\frac{{f}_{o}}{({f}_{o}-{z}_{s})({z}_{d}+{z}_{h})}{\overrightarrow{r}}_{s}]\\  & = & {Q}_{1}[\frac{-{z}_{d}^{2}}{({z}_{d}+{z}_{h})({z}_{d}\,{f}_{d}+{z}_{h}\,{f}_{d}-{z}_{d}{z}_{h})}]\\  &  & \times \,{L}_{1}[-\frac{{f}_{o}}{({f}_{o}-{z}_{s})}(\frac{{f}_{d}}{{z}_{d}\,{f}_{d}+{z}_{h}\,{f}_{d}-{z}_{d}{z}_{h}}{\boldsymbol{E}}-\frac{1}{{z}_{d}+{z}_{h}}{\boldsymbol{R}}(-\theta )){\overrightarrow{r}}_{s}],\end{array}$$

where ***E*** is an identity matrix. Equation (7) is a generalised form expressing the principle of combined 3D imaging and QIDOF imaging. The term *Q*_1_ reflects the axial position of the point source because *z*_*d*_ is a function of *z*_*s*_, according to Eq. (3). The term *L*_1_ determines the transverse magnification of reconstructed images. Thus, the complex amplitude in Eq. (7) is equivalent to a Fresnel hologram. Let *z*_*r*_ denote the distance between the hologram and the reconstruction plane. By applying a numerical back propagation based on the Fresnel diffraction or angular-spectrum method^23^, the image of the point source may be reconstructed at arbitrary planes *z*_*r*_. This means that 3D imaging and numerical refocusing can be implemented. Note that any rotation angle *θ* is applicable to implement 3D imaging. When the focal length *f*_*d*_ of the varifocal lens is set to infinity (i.e. the plane phase distribution), the term *Q*_1_ approaches unity, so Eq. (7) can be rewritten as

8 $${u}_{1}{u}_{2}^{\ast }={L}_{1}[-\frac{{f}_{o}}{({f}_{o}-{z}_{s})({z}_{d}+{z}_{h})}\{{\boldsymbol{E}}-{\boldsymbol{R}}(-\,\theta )\}{\overrightarrow{r}}_{s}].$$

This equation contains no quadratic phase distribution that depends on *z*_*s*_, which results in the loss of axial information about the point source. The complex amplitude in Eq. (8) is equivalent to a Fourier hologram. Thus, an image may be reconstructed by calculating its Fourier transform. In the reconstructed image, as long as a hologram forms on the detector, the point source must be in focus for all axial positions *z*_*s*_, which allows QIDOF imaging to be implemented. This mathematical description shows that both 3D and QIDOF imaging may be implemented in a single setup and that switching between these functionalities requires only changing the parameter *f*_*d*_ but notably does not require mechanical adjustments or any other modifications to the setup. Note that switching the properties of Fresnel and Fourier holograms in a single setup can also be implemented with a Fourier incoherent single-channel holography (FISCH) system^24^. However, in the FISCH system, both Fresnel and Fourier holograms contain 3D information of the object to be captured, which is not applicable to QIDOF imaging. It should also be noted that in order to record digital holograms of general 3D objects, the objects may be represented simply by the incoherent summation of individual digital holograms of point sources. This reconstruction process is linear-shift-invariant and so can be implemented using the same numerical back-propagation method or Fourier-transform method described above.

### Consistency with Previous Studies

To verify the generalised equation, Eq. (7), we now discuss whether it is consistent with previously reported mathematical descriptions of 3D and QIDOF imaging with incoherent digital holography. Although a variety of approaches exist for 3D imaging that leverage the properties of spatially incoherent light sources^25–32^, we mainly focus herein on works related to Fresnel incoherent correlation holography (FINCH)^2,3,33–36^ and image-inverted interferometry^9–11^.

There is no coordinate rotation for conventional 3D imaging with incoherent digital holography^2,3,33–36^, so *θ* = 0° in Eq. (7), and we get

9 $${u}_{1}{u}_{2}^{\ast }={Q}_{1}[\frac{-{z}_{d}^{2}}{({z}_{d}+{z}_{h})({z}_{d}\,{f}_{d}+{z}_{h}\,{f}_{d}-{z}_{d}{z}_{h})}]{L}_{1}[{M}_{T}\frac{-{z}_{d}^{2}}{({z}_{d}+{z}_{h})({z}_{d}\,{f}_{d}+{z}_{h}\,{f}_{d}-{z}_{d}{z}_{h})}{\overrightarrow{r}}_{s}],$$

where *M*_*T*_ is the transverse magnification of the reconstructed image:

10 $${M}_{T}=\frac{{z}_{h}\,{f}_{o}}{{z}_{d}({f}_{o}-{z}_{s})}.$$

Straightforward algebraic manipulations show that these equations are consistent with that of FINCH given in^33–36^.

In contrast, the rotation angle is *θ* = 180° in Eq. (8) for conventional QIDOF imaging^9–11^, which yields

11 $${u}_{1}{u}_{2}^{\ast }={L}_{1}[-\frac{2{f}_{o}}{({f}_{o}-{z}_{s})({z}_{d}+{z}_{h})}{\overrightarrow{r}}_{s}].$$

Moreover, the distance *d* + *z*_*h*_ is set to zero, and removing the lens (i.e. *f*_*o*_ → ∞) yields

12 $${u}_{1}{u}_{2}^{\ast }={L}_{1}[-\frac{2}{{z}_{s}}{\overrightarrow{r}}_{s}],$$

which is consistent with the previously derived equation based on the coherence function or mutual intensity function^9–11^. Moreover, Eqs (8)–(11) are compatible with a previously reported equation based on a cosine transform and a small-angle approximation^37^.

This theoretical analysis implies that the proposed generalised theory is consistent with previous studies. The validity of the generalised equation is experimentally investigated in the following experimental demonstration.

### Telecentricity Conditions

As shown in Eqs (7), (8), (10) and (11), the transverse magnification of reconstructed images depends on the axial position *z*_*s*_ of objects, which leads to possible misunderstandings about the size of real 3D objects. To avoid this problem, we fix the transverse magnification of reconstructed images regardless of *z*_*s*_. When a recording setup is telecentric, the transverse magnification can be fixed. Here we discuss the conditions for telecentricity of 3D and QIDOF imaging.

For 3D imaging, the telecentricity condition is obtained by setting *θ* = 0 and *f*_*o*_ = *d*. Applying these conditions to Eq. (10) yields

13 $${M}_{T}=\frac{{z}_{h}}{{f}_{o}},$$

which is independent of the axial position *z*_*s*_. This transverse magnification has been found in previous studies^35,38,39^.

In contrast, the telecentricity condition for QIDOF imaging can be obtained using *f*_*o*_ = *d* + *z*_*h*_. Equation (8) then takes the following form:

14 $${u}_{1}{u}_{2}^{\ast }={L}_{1}[-\frac{1}{{f}_{o}}\{{\boldsymbol{E}}-{\boldsymbol{R}}(-\,\theta )\}{\overrightarrow{r}}_{s}],$$

which is also independent of the axial position *z*_*s*_. Unlike for 3D imaging, the feasibility of the telecentricity condition for QIDOF imaging has yet to be experimentally verified. Thus, in this work, we experimentally evaluate the effectiveness of the telecentricity condition for QIDOF imaging.

Note that the telecentricity conditions for 3D and QIDOF imaging differ from each other, so it is impossible to satisfy both conditions without modifying the optical setup shown in Fig. 1. Note that when a setup is telecentric, the field of view or the spatial resolution of the reconstructed images for both 3D and QIDOF imaging tends to be relatively small owing to the reduced overlap of interfering beams^12,40^. To satisfy the telecentricity conditions, we must therefore create an optical configuration as a function of the application and the target properties of images.

## Experimental Demonstration

### Experimental Setup

**Figure 2:**
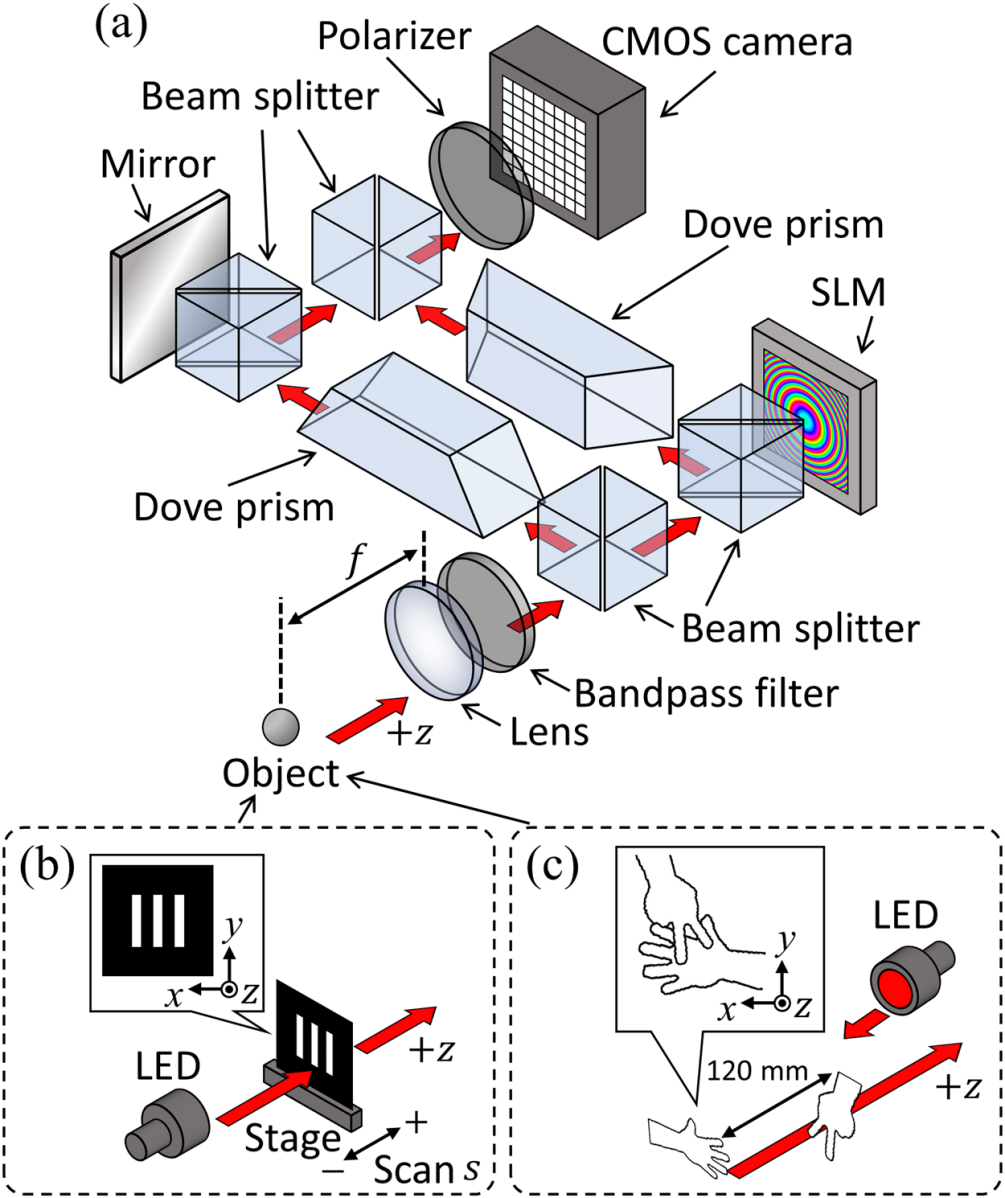
Experimental setup for bimodal incoherent digital holography. (**a**) Recording setup. (**b**) Line-shaped mask for evaluating the DOF. (**c**) Metal plates shaped as ‘scissors’ and ‘paper’ were used to demonstrate 3D and QIDOF imaging.

As a proof-of-principle experiment for bimodal incoherent digital holography, we used the experimental setup shown in Fig. 2. The setup was based on a Mach–Zehnder interferometer. An LED with a centre wavelength of 625 nm and a bandwidth of 18 nm served as a spatially incoherent light source. A 10 nm bandpass filter centred at 633 nm was used to enhance the temporal coherence of the LED. A lens with a 400 mm focal length collected the light. A pair of dove prisms allowed the spatial coordinates of the beam to be rotated. Note that a single dove prism generally reverses a beam and rotates its spatial coordinates, whereas the use of two dove prisms (one in each optical path) eliminates this beam reversal. In previous work, a right-angled prism or a corner cube prism was used instead of the dove prisms to rotate the beam^10,25,37^. However, the ridgeline in prisms leads to a line-shaped artefact in digital holograms. To avoid this potential artefact, we used dove prisms. One of the dove prisms was rotated 90° with respect to the other, which resulted in a relative rotation by *θ* = 180° between the two beams without influencing the polarisation state^41^. A spatial light modulator (SLM) with 1,408 × 1,058 pixels and a pixel pitch of 10.4 μm served as a varifocal lens. The SLM produced a quadratic phase pattern with focal length *f*_*d*_ = 600 mm or a plane phase pattern *f*_*d*_ → ∞, which allowed switching between 3D imaging and QIDOF imaging. Moreover, to implement the four-step phase-shift algorithm^42^ and to eliminate the bias and twin image of a digital hologram, the SLM could introduce four phase shifts (0, *π*/2, *π* and 3*π*/2) on the beam during recording. In the optical path without the SLM, a mirror was placed immediately behind a beam splitter. This configuration imitated that of the optical path including the SLM to match the intensities of two beams reasonably. Moreover, the combination of the mirror and the beam splitter proved useful in minimizing the optical path difference between two beams by solely displacing the mirror. A polariser extracted the phase-modulated polarisation component from the SLM to create an interference pattern without the undesirable orthogonal polarisation component. A 8-bit complementary metal-oxide semiconductor (CMOS) camera with 10,000 × 7,096 pixels and a pixel pitch of 3.1 μm served to capture digital holograms. To experimentally verify the telecentricity condition of QIDOF imaging, the optical configuration was aligned so that *f*_*o*_ = *d* + *z*_*h*_. The lens and the SLM were a distance *d* = 100 mm apart, and the SLM and CMOS camera were separated by *z*_*h*_ = 300 mm. This setup was used to record the digital holograms of two types of objects [see Fig. 2(b,c)].

### Evaluation of DOF

**Figure 3:**
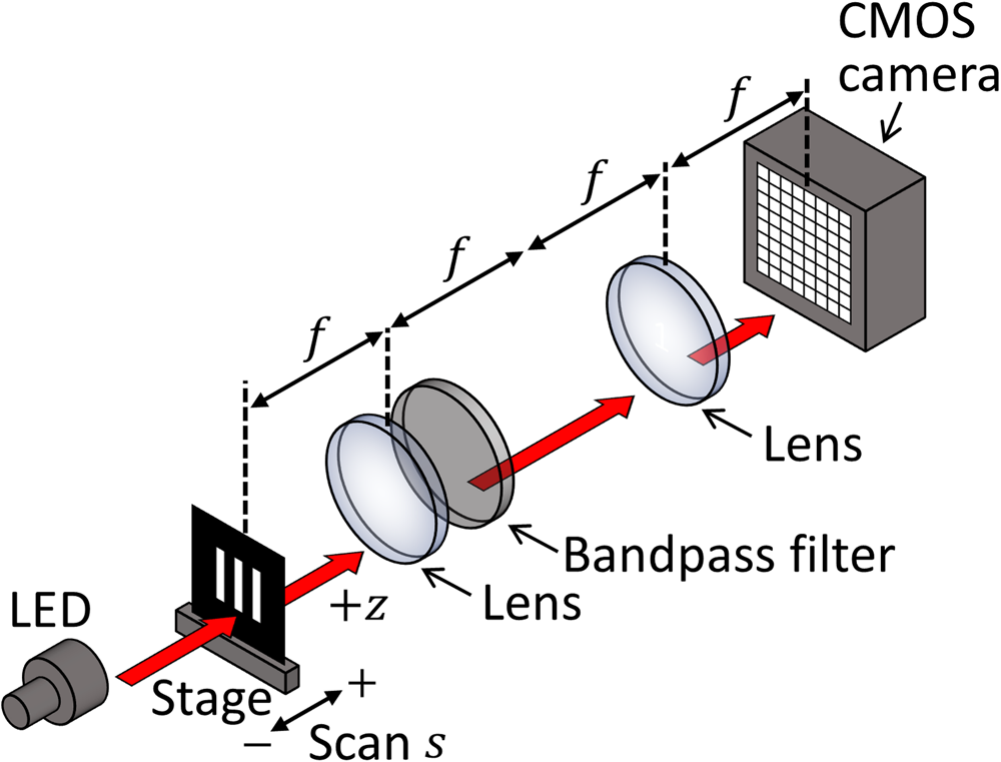
Experimental setup for evaluating the DOF of a conventional imaging system.

To assess the capability of the proposed system for 3D and QIDOF imaging and validate the telecentricity condition for QIDOF imaging derived from the generalised theory, we experimentally evaluated the DOF of each imaging technique by recording and reconstructing digital holograms of the line-shaped mask shown in Fig. 2(b). The mask consisted of three vertical parallel lines, which allowed us to evaluate the DOF simply from the amplitude of its spatial-frequency alternating current (AC) component. The mask was set on a scanning stage and initially positioned near the front focal plane of the lens. The scanning stage was axially scanned at 10 mm intervals from *s* = −100 mm to *s* = +100 mm to emulate objects positioned at different axial planes. The mask at each scanned position was back-illuminated by the LED, and the digital hologram of the transmitted beam was captured. Both the 3D and the QIDOF imaging techniques were evaluated in this way by changing the phase patterns on the SLM. For comparison, we also evaluated the DOF of the conventional 4*f* imaging setup shown in Fig. 3, for which the specifications of the optics are the same as in the proposed setup, so the numerical aperture defined by the object is the same as in Fig. 2(a). Note that the bandpass filter was introduced into the 4*f* imaging setup to evaluate the DOF under the same temporal coherence as the proposed setup.

**Figure 4:**
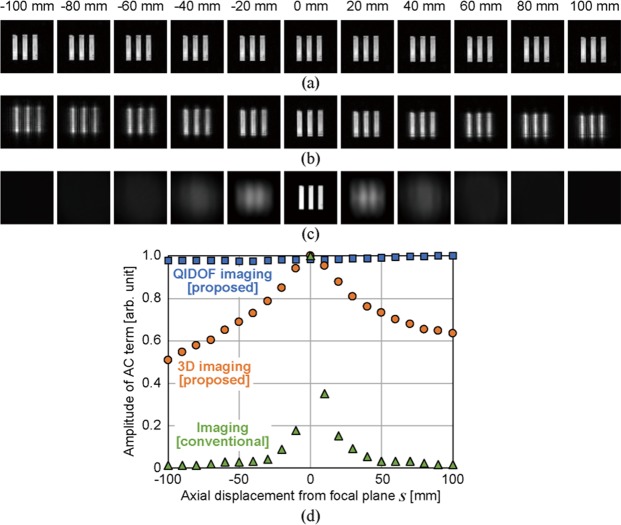
Evaluation of the DOF. Reconstructed images for (**a**) QIDOF imaging and (**b**) 3D imaging in the proposed system and (**c**) images captured by a conventional imaging setup. (**d**) AC amplitude components of each image as a function of the axial displacement from the focal plane.

Figure 4(a–c) show the reconstructed and captured images for each imaging technique. Note that the bokeh of each image differs qualitatively. To quantitatively compare the DOF of each imaging technique, we evaluated the amplitudes of the horizontal AC spatial-frequency component of the images. The blurred image of the three vertical lines means that the AC component of the image should be small, which provides information about the DOF. The amplitude of the AC component of the reconstructed images was extracted as follows: The 2D reconstructed image was segmented into horizontal one-dimensional (1D) arrays of pixels. Subsequently, the 1D Fourier spectrum of each 1D pixel array was obtained individually by calculating the 1D Fourier transform. To mitigate the effect of detector noise, we averaged the amplitude of the 1D Fourier spectra. Finally, the maximum amplitude of the main AC component was extracted so that the evaluation would be independent of the transverse magnification of the images. Figure 4(d) compares the extracted amplitudes of the AC component of each image. These results are consistent with the behaviour of the image bokeh shown in Fig. 4(a–c).

For QIDOF imaging, the reconstructed images are clearly in focus, and the AC amplitude remains almost constant, regardless of the axial displacement, as shown in Fig. 4(a,d). Moreover, the transverse magnification of the images remains constant because of the telecentricity condition for QIDOF imaging, as expected from the theory. Figure 4(b) shows the images reconstructed by the proposed 3D imaging system. Upon applying numerical propagation, the reconstructed images were fixed at the same reconstruction distances that were used to obtain the in-focus image at *s* = 0. When the mask is displaced axially from *s* = 0, the images tend to blur. Note that the transverse magnification of the 3D imaging depends on the axial position of the mask because the optics are aligned to fulfil the telecentricity condition for QIDOF imaging. Figure 4(c) shows the captured images of the conventional 4*f* imaging system. In contrast to the 3D imaging of the proposed method, the images rapidly blur, and the change in the AC component, shown in Fig. 4(d), becomes narrower than that in the image obtained by 3D imaging. In terms of axial resolution, the 3D imaging of the proposed system is inferior to the conventional 4*f* imaging. This relatively wide DOF increases the ambiguity of the axial position of 3D objects, which is known to be a major disadvantage of incoherent digital holography^5^. This drawback can be mitigated by changing the configuration of the optics according to the generalised equation [Eq. (7)], because the DOF is related to the transverse magnification of the images. Alternatively, the DOF can be shortened by introducing diffuser-aided correlation or compressive sensing^22,32,43^.

Based on these qualitative and quantitative comparisons, we verified the effectiveness of the generalised theory and functions of QIDOF and 3D imaging by the proposed system. The experiment described above has been carried out only for transmissive 2D objects. Thus, in the following, we experimentally verify the 3D and QIDOF imaging techniques by using them to image a reflective 3D object.

### Recording and Reconstructing a Reflective Object

To further verify the feasibility of the proposed bimodal incoherent digital holography, we experimentally recorded the image of a reflective object. The objects in question were two metal plates shaped as the scissors and paper of the hand game [see Fig. 2(c)] and separated along the axis by 120 mm. Viewed along the *z*-direction, the metal plates partially overlapped to confirm the occlusion effect. The metal plates were illuminated by an LED, and the reflected light was captured as a digital hologram. While capturing digital holograms, we switched the phase pattern on the SLM to implement either 3D imaging or QIDOF imaging.

**Figure 5:**
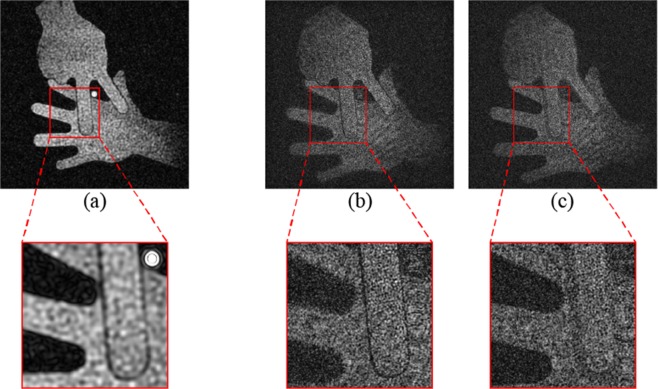
Experimental images of a reflective 3D object obtained using the proposed system. A reconstructed image obtained by (**a**) QIDOF imaging. Reconstructed images obtained by 3D imaging with focus at (**b**) scissors and (**c**) paper.

Figure 5 shows the images reconstructed by QIDOF and 3D imaging. To verify the image detail, an enlarged view is provided for each image in Fig. 5. Note that the transverse magnification differs between reconstructed images in Fig. 5 because of the different reconstruction procedures. To qualitatively compare the reconstructed images, they were resized to be of approximately the same size. In Fig. 5(a), both metal plates are in focus. Moreover, because the metal plates overlap, part of the paper plate is hidden behind the scissors plate. The bright spot at the centre of the image corresponds to the optical axis and is mainly caused by phase-shifting error and detector noise. Because the reconstruction is based on a Fourier transform, the zeroth-order component due to phase-shifting error and detector noise concentrates on the optical axis and forms a bright spot in the reconstruction. Note that this bright spot also appears in Fig. 4(a), although it is displaced with respect to the reconstructed image.

Figure 5(b,c) show the images reconstructed by 3D imaging. The scissors and paper images are in focus individually. The distance between the two focused images was numerically determined to be 110 mm, which differs slightly from the actual configuration. This discrepancy might be caused by aberrations due to the refractive-index mismatch between the air and the optics. Compared with the image reconstructed by QIDOF imaging and shown in Fig. 5(a), the images reconstructed by 3D imaging and shown in Fig. 5(b,c) seem noisy, which may be due to the different propagation distances. The Fourier transform used in the reconstruction by QIDOF imaging may be regarded as propagation over an infinite distance. In contrast, 3D imaging based on Fresnel propagation involves propagation over a finite distance. Thus, in reconstruction by QIDOF imaging, the optical field propagates farther than in reconstruction by 3D imaging. Because of this long propagation distance, the noise component may become randomly diverse, expect for the zeroth-order spot in the image reconstructed by QIDOF imaging. These experimental results show that the proposed system of bimodal incoherent digital holography provides both 3D and QIDOF imaging.

## Conclusion

In this paper, we derived a generalised theory based on wave propagation for 3D imaging and QIDOF imaging with incoherent digital holography. This generalised theory is consistent with previous studies and allows us to comprehensively discuss both imaging techniques, thereby providing significant insights into the properties of each technique. By applying this generalised theory, we reveal, in particular, the different telecentricity conditions for 3D and QIDOF imaging. The generalised theory also allows us to discuss the transverse and axial resolutions and the field of view of each imaging technique, which will be refined in future work. Guided by this generalised theory, we experimentally demonstrate a bimodal incoherent digital holography system.

To verify the capability of the proposed system for 3D imaging and the correspondingly derived equation, we evaluated its DOF and imaged a reflective 3D object. In previous studies on FINCH, holograms of 3D objects were recorded without rotating the coordinate system of the beam. In contrast, in the present work, we recorded holograms with the beam coordinate system rotated. As described by Eq. (7), the transverse magnification depends on the angle through which the coordinate system is rotated. Unlike conventional FINCH, the additional degree of freedom in the proposed system is useful because it allows flexibly changing certain imaging properties, such as spatial resolution, DOF and field of view. However, a more detailed investigation of this point is left for future work.

To verify the capability of the proposed system for QIDOF imaging and the correspondingly derived equation, we demonstrated its feasibility by imaging both a transmissive mask and a reflective 3D object. Moreover, while evaluating the DOF, we experimentally verified that the effectiveness of the telecentricity condition for QIDOF imaging is consistent with that expected from the generalised theory. QIDOF imaging with the telecentricity condition prevents any possible misunderstanding of the size of the real 3D image. In digital holography, an extended DOF is generally obtained by merging many reconstructed images from different axial planes^44^, which, depending on the volume of interest, requires significant calculations of numerical propagation and results in a time-consuming process. Although this problem has been mitigated by the introduction of deep learning^45^, such a technique is invalid for 3D objects outside of the training region. In contrast, QIDOF imaging with incoherent digital holography delivers images with extremely large DOF simply by calculating a single Fourier transform, with no training.

The above preliminary experimental results prove the effectiveness of the derived equations for bimodal incoherent digital holography. The proposed bimodal incoherent digital holography system thus provides both 3D imaging and QIDOF imaging techniques, which provides additional information on real 3D scenes and objects in many applications.
